# Aiming for precision: personalized medicine through sepsis subtyping

**DOI:** 10.1093/burnst/tkae073

**Published:** 2025-01-03

**Authors:** Aryna Kolodyazhna, W Joost Wiersinga, Tom van der Poll

**Affiliations:** Amsterdam University Medical Center, University of Amsterdam, Center of Experimental and Molecular Medicine & Division of Infectious Diseases, Amsterdam Institute for Infection and Immunity, Amsterdam, the Netherlands; Amsterdam University Medical Center, University of Amsterdam, Center of Experimental and Molecular Medicine & Division of Infectious Diseases, Amsterdam Institute for Infection and Immunity, Amsterdam, the Netherlands; Amsterdam University Medical Center, University of Amsterdam, Center of Experimental and Molecular Medicine & Division of Infectious Diseases, Amsterdam Institute for Infection and Immunity, Amsterdam, the Netherlands

**Keywords:** Sepsis, Subtyping, Immune response

## Abstract

According to the latest definition, sepsis is characterized by life-threatening organ dysfunction caused by a dysregulated host response to an infection. However, this definition fails to grasp the heterogeneous nature and the underlying dynamic pathophysiology of the syndrome. In response to this heterogeneity, efforts have been made to stratify sepsis patients into subtypes, either based on their clinical presentation or pathophysiological characteristics. Subtyping introduces the possibility of the implementation of personalized medicine, whereby each patient receives treatment tailored to their individual disease manifestation. This review explores the currently known subtypes, categorized by subphenotypes and endotypes, as well as the treatments that have been researched thus far in the context of sepsis subtypes and personalized medicine.

Highlights

- The highly heterogeneous nature of sepsis arises from various factors, including diverse causative pathogens and individual patient characteristics such as age, sex, and comorbidities. This variability complicates treatment and suggests that a one-size-fits-all approach may be ineffective.
- Recent studies have shown that sepsis patients can be stratified into more homogeneous subgroups, based on clinical characteristics (subphenotypes) or underlying pathophysiological mechanisms (endotypes).
- Identification of subgroups among sepsis patients may enhance the understanding of patient outcomes and guide personalized medicine approaches. Nonetheless, challenges remain in establishing standardized criteria for classification and ensuring that diagnostic methods are practical for clinical use.

## Background

Considering the most recent International Consensus Definition of Sepsis (Sepsis 3.0), outlined in 2016, sepsis ought to be characterized as a state of life-threatening organ dysfunction caused by a dysregulated host response to infection [1]. Septic shock is a subset of sepsis, described by persistent hypotension requiring vasopressors to maintain a sufficient mean arterial pressure and adequate tissue perfusion, that is associated with higher mortality rates [1]. In 2017, the worldwide incidence of sepsis was estimated at 48.9 million cases, with 11.0 million reported sepsis-related deaths, representing a considerable 19.7% of all global deaths [2]. While the incidence fell by 37.0% and mortality decreased by 52.8% from 1990 to 2017, mostly due to antibiotic therapy and supportive care, the numbers remained stagnant since 2011 [2–4]. Currently available treatments for sepsis consist of source control, timely antibiotics, resuscitation, and supportive care for organ dysfunction, yet do not target specific host response aberrations [5]. Despite the significant number of randomized controlled trials (RCTs) conducted in the last decades, there remains a lack of effective treatments that show significant improvements in outcomes among sepsis patients [6,7]. In fact, some RCTs found that the intervention’s observed effect on outcomes was opposite to the anticipated effect [8]. This lack of success has been attributed for a large part to the highly heterogeneous nature of sepsis, finding its origin in a broad range of causative pathogens (e.g. bacterial, viral, fungal, protozoal, parasitic), together with differences in comorbidities, sex, age, (epi)genetics, and environmental factors, resulting in a significant variability in host responses [9,10]. In this context, it has been proposed that stratification of sepsis patients into more homogeneous subgroups could be the answer to developing novel effective therapies [3,11,12]. Stratification or “phenotyping” diseases is a novel development within the medical field, made possible by recent improvements in computational power combined with the availability of high-throughput data known as “omics” [13,14]. Subgrouping sepsis can be approached from various perspectives and following different methodologies. First, subgrouping of patients can be done based on measurable characteristics that inform treatment responses (predictive enrichment) or outcomes (prognostic enrichment) [15,16]. Additionally, subgrouping can also be based on common clinical and laboratory features (subphenotypes), as opposed to subgrouping based on the underlying pathobiological abnormalities (endotypes) [17]. This approach may lead to the opportunity to match the right therapies to the right patients at the right time, also known as precision medicine. Precision medicine has already demonstrated promising results in other medical fields such as asthma and oncology, but remains in its infancy in the field of sepsis [9,15,18,19]. This narrative review explores the current understanding of the diverse subtypes of sepsis that have been researched so far and their potential applications in innovative therapeutic approaches.

**Table 1:** Overview of subphenotypes

**Subphenotype (prevalence)**	**Main characteristics**	**Mortality (%)**	**Ref**
***SENECA subphenotypes*** K-means clustering (29 clinical and laboratory variables)			21
α (33%)	Less organ dysfunction	2^a^	
β (27%)	Older patients, more chronic illness, renal dysfunction	5	
γ (27%)	More inflammation, higher temperature	15	
δ (13%)	More organ dysfunction, higher lactate, more hypotension	32	
***Temperature trajectory subphenotypes*** Group-based trajectory modeling (temperatures over the first 72 h)			22
Hyperthermic, slow resolvers (14.9%)	Youngest group, fewest comorbidities, highest inflammatory markers	5.1^b^	
Hyperthermic, fast resolvers (23.2%)	Fastest temperature resolution	2.9	
Normothermic (32.8%)	Intermediate characteristics between other groups	5.3	
Hypothermic (29.1%)	Oldest group, most comorbidities, lowest inflammatory markers	9.5	
***Organ dysfunction subphenotypes*** Latent profile analysis (clinical and laboratory variables)			23
Profile 1—Baseline type (69%)	Lowest SOFA score, longest hospital length of stay	16.9^b^	
Profile 2—Respiratory dysfunction (9%)	Low PaO_2_ and high PaCO_2_	18.2	
Profile 3—Multiple organ dysfunction (11%)	Highest SOFA score, with kidney, coagulation, liver, and shock dysfunction, and lowest blood pressure	45.4	
Profile 4—Neurological dysfunction (11%)	Low Glasgow Coma Scale score	27.4	
***Organ dysfunction subphenotypes*** Kohonen self-organizing maps and K-means clustering (clinical variables)			24
Cluster 1 (20.6%)	Shock with elevated creatinine	11^c^	
Cluster 2 (45.1%)	Minimal multi-organ dysfunction syndrome	12	
Cluster 3 (22.0%)	Shock with hypoxia and altered mental state	28	
Cluster 4 (12.2%)	Hepatic disease	21	
***Sepsis classes*** Latent class analysis (46 clinical variables)			25
Class 1 (18.3%)	Uncomplicated septic shock with few organ dysfunctions	23.6^d^	
Class 2 (22.0%)	Pneumonia with ARDS	27.4	
Class 3 (19.6%)	Postoperative abdominal	31.3	
Class 4 (18.8%)	Severe septic shock, high lactate, low platelet counts, highest likelihood of bloodstream infection	41.2	
Class 5 (17.1%)	Pneumonia with ARDS and multiple organ dysfunction syndrome	48.6	
Class 6 (4.2%)	Late septic shock	52.1	
***Subphenotypes in early septic shock*** Hierarchical clustering, with consensus clustering (23 clinical variables)			26
L1 (28%)	Fluid-responsive septic shock	9.6^a^	
L2 (21%)	Fluid-refractory septic shock and multiple organ failure	14.1	
M (16%)		32.4	
H1 (19%)	Oldest group, multi-organ dysfunction with highest SOFA scores	34.4	
H2 (16%)	Youngest group, liver failure and coagulopathy	41.2	

Mortality at day 28^a^, day 30^c^, day 90^d^, or in-hospital^b^

## Review

### Subphenotypes

The term “subphenotype” refers to a homogeneous group distinguished by a specific set of features not necessarily linked by a common pathophysiological mechanism, but rather by overlapping clinical characteristics [15,20]. Subphenotypes can be identified using readily available clinical and routine laboratory data. Following this approach, multiple diverse subphenotypes have been reported to date (Table 1). Four different clinical subphenotypes were identified with data obtained from the emergency department in the SENECA study: α subphenotype (33%, less organ dysfunction, mortality 2%); β subphenotype (27%, more chronic illness and renal dysfunction, mortality 5%); γ subphenotype (27%, more inflammation and higher temperature, mortality 15%); δ subphenotype (13%; higher lactate and more hypotension, mortality 32%) [21]. Bhavani *et al.* categorized sepsis patients into four subphenotypes based on their temperature trajectory: “hyperthermic, slow resolvers” (14.9%), “hyperthermic, fast resolvers” (23.2%), “normothermic” (32.8%), and “hypothermic” (29.1%) [22]. The hypothermic subphenotype entailed the oldest patients with the most comorbidities, combined with the lowest levels of inflammatory markers and the highest in-hospital mortality rate (9.5%). The hyperthermic, slow resolvers were the youngest with the fewest comorbidities, the highest levels of inflammatory markers, and a mortality rate of 5.1%. The hyperthermic, fast resolvers had the lowest mortality rate (2.9%). Zhang *et al.* identified four subphenotypes classified by type of organ dysfunction (e.g. respiratory, neurological, multiple organ dysfunction), each displaying distinct mortality outcomes in correlation to fluid resuscitation [23]. Profile 3, characterized by multiple organ dysfunction (kidney, coagulation, liver, and shock), exhibited the highest mortality rate (45.4%), followed by profile 4 (27.4%), characterized by neurological dysfunction. While increased fluid input reduced the risk of hospital mortality for profile 3, it conversely heightened the mortality for profile 4. Correspondingly, Knox *et al.* identified four clusters using age and Sequential Organ Failure Assessment (SOFA) subscores: (1) shock with elevated creatinine; (2) minimal multi-organ dysfunction syndrome; (3) shock with hypoxemia and altered mental status; (4) hepatic disease [24]. Another study identified six subphenotypes using clinical assessments and laboratory data in a similar manner: (1) “uncomplicated septic shock”; (2) “pneumonia with adult respiratory distress syndrome (ARDS)”; (3) “postoperative abdominal”; (4) “severe septic shock”; (5) “late septic shock” [25]. Class 1 (“uncomplicated septic shock”) patients displayed few organ dysfunctions and low SOFA scores, originating from otherwise uncomplicated infections such as urinary tract, lung, and skin infections. In contrast, class 4 (“severe septic shock”) patients showed the highest likelihood for positive blood cultures and often had relatively high lactate and low platelet counts. These patients also frequently underwent steroid treatment for sepsis.

A retrospective analysis of the ProCESS trial identified five phenotypes based on organ failure patterns that persisted in time [26]. The high-risk phenotypes, H1 and H2, exhibited the highest mortality at both 14 days (28.8%; 36.3%) and 60 days (42.4%; 44.1%). H1 was present in 19% of the patients and constituted the eldest subgroup characterized by the highest baseline Acute Physiology and Chronic Health Evaluation and SOFA scores. H1 was further defined by multi-organ dysfunction, particularly cardiac (81.6%) and respiratory (59.2%) failure [26]. H2, observed in 16% of the subjects, comprised the youngest subgroup and exhibited a unique pattern of organ dysfunction consisting of liver failure and coagulopathy, alongside a higher likelihood for intra-abdominal infection and for a positive blood culture, with nearly half of the cases manifesting bacteremia [26]. This phenotype also exhibited the highest likelihood of developing new renal failure (13.7%) and new cardiovascular failure (78.3%), while also demonstrating the highest rates of pre-existing renal failure (26.5%) and chronic liver disease (30.4%) [26]. Furthermore, liver dysfunction and coagulopathy persisted even at 72 h post-onset. Importantly, the high-risk phenotypes displayed distinct trajectories over time, with H2 maintaining elevated bilirubin levels, decreased platelet counts, elevated international normalized ratio (INR), and a trend toward persistent shock at 7 h, while a notable proportion of H1 had already shown signs of recovery by that time [26]. The remaining phenotypes were fluid-responsive septic shock (L1, low risk), fluid-refractory septic shock with multi-organ failure (L1, low risk), and septic shock with respiratory failure (M, moderate risk) [26].

As subphenotypes essentially represent clinical subclasses of sepsis, they enable the inclusion of data from millions of patients to identify robust sepsis subclasses for estimating clinical outcomes [20,21,27]. Nevertheless, inclusion based on clinical data also represents its own set of challenges, as clinical definitions such as sepsis, infection, and acute organ dysfunction often have unclear boundaries, possibly resulting in variability in patient inclusion based on the researcher and/or clinician [20,28]. Likewise, organ dysfunction (e.g. kidney disease, cardiovascular dysfunction, coagulopathy) can be pre-existing to sepsis, posing challenges in distinguishing whether the observed changes are attributed to the present infection, the progression of the chronic condition, or a combination of both [20,29]. On the same note, while host response biomarkers may suggest the potential underlying biological mechanism driving these phenotypes, clinical data alone offer limited insight into the intrinsic processes, potentially lacking precision [20,21].

In summary, multiple studies described sepsis subphenotypes based on clinical characteristics and routine laboratory values. The diverse range of parameters and clustering techniques used in these investigations hinders effective inter-study comparison. Consensus on parameters and clustering methods, as well as comprehensive validation, are needed before sepsis subphenotyping can be implemented in clinical practice.

### Endotypes

Contrary to subphenotypes, endotypes are distinguished by specific pathophysiological mechanisms underlying the clinical manifestations, clustering patients who exhibit a shared intrinsic biology driving their sepsis presentation [15,30]. Subgrouping sepsis based on endotypes has experienced exponential growth due to advancements in genomics, transcriptomics, proteomics, and metabolomics, as well as the availability of extensive data sets combined with novel data analysis tools [12,31]. Currently, subgrouping based on endotypes is mainly based on machine learning combined with several clustering techniques, utilizing data obtained from genome-wide transcriptional profiling of whole blood (Table 2).

**Table 2:** Overview of endotypes

**Endotype (prevalence)**	**Main characteristics**	**Mortality (%)**	**Ref**
***Sepsis Response Signatures (SRS)*** ^***a***^ Unsupervised hierarchical cluster analysis for the top 10% most variable probes			33,35
SRS1 (41%)	Immunosuppression, endotoxin tolerance, T-cell exhaustion, and metabolic derangement	22%^a^	
SRS2 (59%)	Immunocompetent, reference endotype	10%^a^	
***Molecular Diagnosis and Risk Stratification of Sepsis (MARS) endotypes*** ^***a***^ Unsupervised consensus cluster analysis			37
MARS1 (29.4%)	Enhanced heme biosynthesis with impaired pattern recognition, cytokine and lymphocyte signaling, and antigen presentation	39%^b^	
MARS2 (34.3%)	Enhanced pattern recognition and cytokine signaling with increased NF-κB and IL-6 signaling	22%^b^	
MARS3 (23.2%)	Enhanced adaptive immune functions (T-cell pathways)	23%^b^	
MARS4 (13.1%)	Enhanced pattern recognition and cytokine signaling with increased interferon signaling	33%^b^	
***Sweeney endotypes*** ^***a***^ Unsupervised unified cluster analysis			36
Inflammopathic (25%)	Innate immune activation	30%^c^	
Adaptive (31%)	Adaptive immune activation	8%^c^	
Coagulopathic (15%)	Clinical and molecular evidence of coagulopathy	25%^c^	
***Calfee endotypes*** ^***b***^ Latent class analysis			40
Hypoinflammatory (70.5%)	Lower inflammatory biomarkers	16.5%^b^	
Hyperinflammatory (29.5%)	Higher plasma pro-inflammatory cytokines, lower protein C, more vasopressor use, more bacteremia	42.6%^b^	
***Early sepsis endotypes*** ^***a***^ Machine learning and data mining			38
Neutrophilic suppressive/NPS (31%)	Immunosuppressed; poorer prognosis, elevated neutrophil proportions, downregulation of inflammatory markers and adaptive signaling pathways	46%^b^	
Inflammatory/INF (17%)	Severe trajectory; upregulation of multiple inflammatory pathways	26%^b^	
Innate host defense/IHD (21%)	Innate host defenses, moderate upregulation of interleukin signaling	0%^b^	
Interferon/IFN (22%)	High expression of interferon-α, -β, and -γ signaling	0%^b^	
Adaptive/ADE (9%)	Mild progression; upregulation of adaptive immune pathways, more abundant lymphocytes	–	

Table summarizes only studies that examined adult patients

Mortality at day 14^a^, 28^b^, or 30^c^

^a^Endotypes based on blood transcriptomes

^b^Endotypes based on clinical parameters and plasma protein biomarkers

Wong and colleagues were the first to describe distinct endotypes in patients with sepsis based on blood transcriptomes [32]. They identified three subphenotypes, termed subclasses A, B, and C, in 98 children admitted to the intensive care unit (ICU) with septic shock. Subclass A had the highest severity of disease and mortality, and was characterized by downregulation of genes related to adaptive immunity, glucocorticoid receptor signaling, and zinc homeostasis/metabolism.

In adults, Davenport *et al.* used transcriptomics analysis of peripheral blood leukocytes from sepsis patients with community-acquired pneumonia to identify two distinct sepsis response signatures (SRS): SRS1 (41%) exhibited a relatively immunosuppressed state characterized by endotoxin tolerance, T-cell exhaustion, and downregulation of human leukocyte antigen (HLA) class II, correlating with higher mortality rates [33]. Subsequently, Kwok *et al.* reported that SRS1 is associated with disturbances in myelopoiesis, leading to increased levels of immature neutrophils and suppression of CD4^+^ T cells, contributing to the immunocompromised characteristics of SRS1 [34]. Contrarily, SRS2 (59%) was described as relatively immunocompetent [33]. The SRS endotypes were also found in sepsis originating from fecal peritonitis [35]. Sweeny *et al.* also identified subclasses through unsupervised analysis of blood transcriptomics: “inflammopathic” (characterized by higher mortality and innate immune activation), “adaptive” (exhibiting lower mortality and adaptive immune activation), and “coagulopathic” (associated with higher mortality, older patients, and clinical and molecular evidence of coagulopathy) [36].

Scicluna *et al.* identified four molecular sepsis endotypes based on blood transcriptomes obtained upon admission to the ICU: Mars1–4, each corresponding to a distinct 28-day mortality [37]. Mars1 was associated with the worst outcome, with a 28-day mortality of 39% compared to the other endotypes: Mars2 (22%), Mars3 (23%), and Mars4 (33%). The Mars1 endotype was characterized by a significant decrease in gene expression corresponding with innate and adaptive immune cell functions, e.g. Toll-like receptor signaling, nuclear factor κB signaling, antigen presentation, and T-cell receptor signaling, combined with an increased expression of specific metabolic pathway genes that included heme biosynthesis [37]. The Mars3 endotype, which was relatively low risk, had an increased expression of adaptive immune or T-cell functions [37]. Employing similar techniques on blood transcriptomes and clinical data collected from patients admitted to the emergency room, Baghela *et al.* identified five distinct endotypes [38]. Based on ~200 distinct gene expression differences and pathways, these patients with “early sepsis” were categorized into the following endotypes: “neutrophilic suppressive/NPS,” “inflammatory/INF,” “innate-host defense/IHD,” “interferon/IFN,” and “adaptive/ADA” [38]. Each endotype was associated diverse outcomes, with NPS/INF corresponding to a severe trajectory compared to ADA, which was associated with a relatively mild disease progression [38].

Two phenotypes of ARDS, termed hyperinflammatory and hypoinflammatory, have been identified using latent class analysis applied to clinical and plasma protein biomarker data [39]. Recently, these ARDS phenotypes were also found in patients with sepsis, endorsing the notion that critical illness in general can be stratified in more homogeneous subgroups based on host response characteristics irrespective of the inciting insult (e.g. infection or major trauma) [40,41]. Across different cohorts, approximately one-third of sepsis patients were classified as hyperinflammatory and two-thirds as hypoinflammatory. The hyperinflammatory endotype was associated with higher plasma pro-inflammatory cytokines, more vasopressor use, lower protein C, and higher mortality, as compared with the hypoinflammatory endotype [40]. In additional analyses, the hyperinflammatory and hypoinflammatory phenotypes of sepsis were found to have distinct blood gene expression signatures, with 5755 genes (31%) differentially expressed [42]. The hyperinflammatory phenotype was characterized by enhanced expression of innate immune response genes, while the hypoinflammatory phenotype demonstrated increased expression of adaptive immune response genes. Notably, these phenotypes partially overlapped with the SRS1 and SRS2 endotypes [42].

Reduced monocyte (m)HLA-DR expression is considered a key feature of immunosuppression in patients with sepsis [43]. Bodinier *et al.* investigated the first-week evolution of mHLA-DR surface protein expression in sepsis patients [44]. The following mHLA-DR trajectory endotypes were identified: “non-improvers,” “decliners,” “improvers,” and “high expressors.” The “improvers” and “high expressors” endotypes exhibited a better prognosis in terms of ICU stay, exposure to invasive devices, mortality, and ICU-acquired infections compared to the “non-improvers” and “decliners” endotypes, which were associated with poorer outcomes [44]. Overall, more than half (59%) of the sepsis patients were at risk of deterioration due to low surface mHLA-DR expression, implicating immunosuppression in an adverse sepsis outcome [44].

Plasma concentrations of cytokines and other inflammatory mediators have not been used extensively to identify distinct sepsis clusters. One study entailing sepsis patients with proven bacterial infection without immunosuppression identified three subgroups based on unsupervised hierarchical clustering of 35 inflammatory mediators measured in plasma; while the relation with clinical outcomes was not reported, the cluster with the lowest mediator levels was associated with a lower frequency of patients with bacteremia [45].

Overall, it can be concluded that endotypes characterized by an impaired innate or adaptive immune response are indicative of a poor prognosis, whereas the endotypes that exhibit activation of adaptive immunity demonstrate improved survival chances [46]. However, endotypes displaying an overactive immune reaction, such as the “inflammopathic” and “coagulopathic” endotypes, were also associated with worse outcomes [36,46]. While the stratification of sepsis patients based on endotypes appears to be promising for future research and the identification of new therapeutic targets, along with their clinical application to predict prognosis and triaging, there are still numerous challenges that need to be addressed first. Firstly, it should be considered that the techniques (omics) needed for endotyping within a clinical context should be performed in near-real time, at a reasonable cost, with minimal complexity, and be reproducible [20]. Fortunately, this appears to be achievable in the near future with numerous continuous advancements within the field, resulting in the increasing affordability and accessibility of real-time RNA, protein, and metabolite quantification [20]. Secondly, as evident from the numerous distinct subphenotypes and endotypes discussed in this review, there is no consensus on which subtype(s) should be considered the “golden standard” in sepsis stratification. This lack of consensus contributes to substantial heterogeneity within this research field, potentially complicating the integration of data from separate trials for the development of novel treatments and diagnostic tools. As example, a comparative analysis of several currently available subtypes based on clinical data (α, β, γ, and δ), biomarker data (hyper- and hypoinflammatory), and transcriptomic data (Mars1–Mars4 and SRS1–SRS2) revealed no clear association among the subtyping approaches [47]. This suggests that these subtypes do not identify comparable patient populations and are likely to reflect divergent clinical characteristics and underlying biological mechanisms [47]. The fact that sepsis itself is a dynamic entity complicates subclassification even further, as an individual patient may transition between multiple subclasses over time [1,20]. Hence, each “subtype approach” can possibly yield different subgroupings despite being derived from the same patient population.

### Toward precision medicine and therapeutic heterogeneity

Subtyping of sepsis patients may inform treatment decisions, selecting patients who are more likely to benefit or be harmed by certain therapies, thereby paving the way for precision medicine. The following studies used, at times, pre-established subphenotypes or endotypes of sepsis to investigate options for personalized (immuno-)therapy.

The subphenotypes derived from the SENECA study described above may respond differently to treatments that seek to modify the host response [21]. Monte Carlo simulations were used to artificially change subphenotype distributions in the sepsis populations enrolled in distinct randomized controlled trials. For example, while the ACCESS trial found no effect on mortality after administration of the Toll-like receptor-2/MD2 antagonist Eritoran [48], simulations that increased the δ subphenotype suggested harm from this treatment [21]. Likewise, increasing the proportion of the α subphenotype in simulations of the PROWESS trial suggested no benefit of recombinant activated protein C [21], while the original trial showed a survival advantage [49]. The largest alterations were found in the ProCESS trial, which found no benefit from early goal-directed therapy (EGDT) compared with usual care [50]. Yet, in simulations that increased the proportion of the δ subphenotype, EGDT was harmful in more than half of the simulated trials, while increases in the α subphenotype suggested benefit [21].

Anticliffe *et al.* performed a *post hoc* analysis of the VANISH trial, a double-blind RCT in which patients with septic shock were randomized to receive vasopressin or norepinephrine, followed by hydrocortisone or placebo [51]. In the overall study population, mortality rates did not differ between treatment groups [52]. The *post hoc* analysis tested the hypothesis that this lack of treatment effect was due to heterogeneity within the patient cohort. Consequently, Anticliffe *et al.* divided a subgroup of patients from which blood transcriptomes were available into two SRSs: SRS1 corresponding to the immunosuppressed phenotype associated with increased mortality and SRS2 representing the immunocompetent phenotype [51]. Surprisingly, this approach revealed that hydrocortisone use was associated with increased mortality in the immunocompetent SRS2 phenotype [51]. This effect might be explained by the capability of corticosteroids to downregulate MHCII expression [53,54], and impact NF-κB signaling, T-cell function, and apoptotic pathways, potentially undermining the protective advantages conferred by the SRS2 phenotype [55,56]. Of interest, an independent study indicated that corticosteroids might have a detrimental effect in sepsis patients with the hypoinflammatory endotype originally identified in ARDS [42]. This heterogeneity in treatment response may in part explain earlier conflicting results from previous RCTs, which demonstrated inconsistent effects of hydrocortisone on survival in patients with septic shock [57,58].

The PROVIDE trial prospectively investigated novel therapeutic agents within two subtypes of sepsis defined by single biomarker readouts. Patients were stratified as either having macrophage activation-like syndrome (MALS) [59], a hyperinflammatory syndrome defined by very high ferritin levels, or being immune suppressed, characterized by diminished mHLA-DR expression [60]. Patients classified as MALS were randomized to treatment with anakinra (interleukin (IL)-1 receptor antagonist) or placebo, patients classified as immune suppressed to treatment with recombinant interferon-gamma (IFNγ) or placebo. The trial was stopped prematurely due to slow enrollment rates, yet yielded interesting results. The administration of anakinra resulted in short-term improvements in MALS patients: by day 7 (the end of anakinra administration), anakinra treatment was associated with a decrease in SOFA scores relative to placebo treatment. Anakinra did not impact mortality by day 28, potentially attributable to discontinuation while the hyperinflammatory state still persisted [60]. A treatment effect of IFNγ could not be assessed since only one patient classified as immune suppressed was treated with this immune enhancing cytokine due to an (in retrospect) inadequate selection criterion (<30% expression of mHLA-DRA) [60]. The PROVIDE trial was followed by the larger ImmunoSep (Personalized Immunotherapy in Sepsis) trial with a similar setup, yet with an adapted inclusion criterion for immunosuppression (<5000 HLA-DR molecules on circulating monocytes) [61]. The ImmunoSep trial was completed early 2024; the results are expected later this year.

The ProCESS trial examined the effect of EGDT in patients with septic shock [50]. Patients were randomly assigned to one of three treatment groups: protocol-based EGDT, protocol-based standard therapy, or usual care, wherein protocol-based EGDT prompted placement of a central venous catheter to monitor pressure and to administer intravenous fluids, vasopressors, dobutamine, or packed red-cell transfusions, as directed. In the overall study population, clinical outcomes were similar between treatment arms [50]. From a subgroup of these patients plasma biomarker data [IL-6, intercellular adhesion molecule (ICAM), plasminogen activator inhibitor-1] were available and used to (retrospectively) stratify patients in two phenotypes based on latent class analysis [62]. Phenotype 1 (12% of patients), characterized by increased IL-6, ICAM and total bilirubin, and decreased platelet counts, exhibited increased inflammation, organ dysfunction, and a higher 60-day inpatient mortality compared to phenotype 2 (88% of patients) [62]. Treatment with EGDT was associated with worse 60-day mortality compared to usual care (58% vs. 23%) in phenotype 1, while EDGT did not impact mortality in phenotype 2 (16% vs. 17%) [62]. This retrospective study strongly suggests that the effect of sepsis resuscitation strategies vary according to phenotype, thereby highlighting the need for individualized approaches per patient and/or timeframe.

Sinha *et al.* re-analyzed the results of the PROWESS-SHOCK trial, which determined the effect of recombinant human activated protein C in patients with septic shock [63], after stratification of patients into the hypoinflammatory and hyperinflammatory endotypes, which originally had been identified in patients with ARDS [40]. Remarkably, treatment with activated protein C was associated with higher mortality in the hypoinflammatory endotype and lower mortality in the hyperinflammatory endotype compared with placebo.

The SCARLET trial examined the effect of recombinant human soluble thrombomodulin in patients presenting with sepsis-associated coagulopathy [64]. In the trial, no significant improvement in mortality was observed with the administration of human recombinant thrombomodulin [64]. However, in an observational study based on registry data conducted in Japan (where thrombomodulin is a registered drug), unsupervised clustering analysis using a set of readily available coagulation parameters identified a subgroup of patients with a high likelihood of survival benefit, showing treatment heterogeneity even within a cohort of patients with sepsis-associated coagulopathy [65].

Similarly focused on a specific condition in sepsis, the REVIVAL trial investigated the therapeutic effect of ilofotase alfa (human recombinant alkaline phosphatase) for sepsis-associated acute kidney injury (SA-AKI) [66]. In a retrospective analysis, patients were divided into two phenotypes based on a stepwise machine learning approach: phenotype 1, characterized by relatively lower disease severity and less pronounced renal and pulmonary dysfunction; and phenotype 2, which exhibited higher severity scores and creatinine, with lower estimated glomerular filtration rate and reduced bicarbonate levels [67]. Compared to the placebo treatment, ilofotase alfa administration was associated with a significant reduction in Major Adverse Kidney Events up to day 90 (MAKE90) in phenotype 2 patients, and not in phenotype 1 patients [67]. These results show the potential for enhanced therapeutic options tailored to this specific patient group and thereby potentially advancing personalized medicine in SA-AKI.

**Figure 1:**
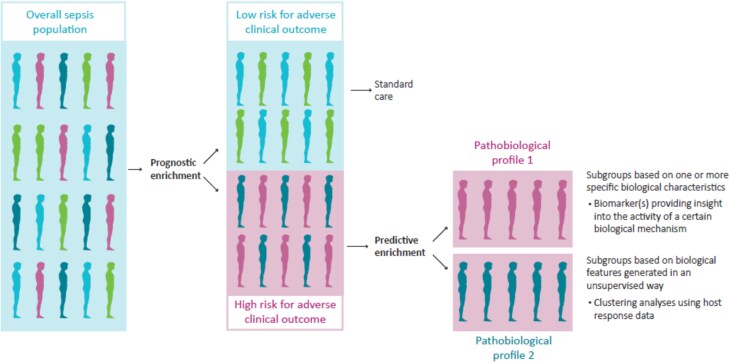
Implementation of precision medicine in sepsis. The current definition of sepsis captures a highly heterogeneous patient population with distinct clinical outcomes and underlying pathophysiological mechanisms. The vast majority of sepsis trials performed to date enrolled patients based solely on severity of disease (e.g. the presence of shock), thereby enriching the population for patients who are more likely to reach the primary outcome (e.g. mortality). This approach ignores underlying pathobiological mechanisms targeted by the therapeutic tested in the trial. Predictive enrichment seeks to enrich the population for patients with certain pathobiological profiles, i.e. more homogeneous subgroups that are more likely to respond to specific host response interventions. Biological profiling could be done by a limited set of biomarkers (e.g. ferritin for hyperinflammation and mHLA-DR for immunosuppression) or by unsupervised cluster analyses using large host response data sets (e.g. blood transcriptomics)

In summary, retrospective analyses of trial data indicate that novel treatments that target the host response in patients with sepsis, while ineffective in the overall trial population, might be beneficial in subgroups of patients, identified by specific biomarkers (e.g. mHLA-DR) or unsupervised clustering methods (phenotypes and endotypes). Personalized medicine holds promise, wherein the application of prognostic enrichment of the population (i.e. selecting patients at high risk for an adverse clinical outcome) combined with predictive enrichment (i.e. selecting patients with a high likelihood of a treatment effect based on their pathobiological profile) is an attractive approach to move forward (Figure 1).

### Remaining challenges

Although significant progress has been made in the understanding of the pathophysiology of sepsis, this has not been translated into specific treatments yet. Patient stratification based on clinical and/or pathobiological features holds promise for precision medicine in sepsis, targeting patients who might benefit from a therapeutic and withholding a drug or intervention in those who are unlikely to benefit or can even be harmed. In the past few years, many studies have been published that used different techniques to generate subgroups of sepsis patients with more similar clinical and/or biological characteristics. Retrospective analyses of RCTs indeed suggest that *a priori* stratification of patients may reveal novel therapies for sepsis that were deemed ineffective based on overall RCT results. A major weakness in studies that reported sepsis subgroups thus far is the lack of a uniform approach, making comparisons between investigations difficult. A critical next step is to establish a consensus regarding the sepsis subclassification systems, integrating clinical data with underlying transcriptomic and other intrinsic signatures. To achieve this, a global collaboration among the various investigator groups is needed, together with the validation of both existing datasets and prospective studies. Another issue that deserves attention is that currently described sepsis subtypes are primarily based on singular measurements, thereby ignoring that sepsis is a dynamic entity evolving over time [22,44,62,68]. Therefore, it is crucial to obtain more time-dependent information about sepsis subphenotypes and endotypes. In this regard, serial sampling up to day 5 after ICU admission revealed that SRS group membership changed in 46% of patients, of whom the majority (90%) moved from SRS1 to SRS2 [36]. Likewise, a study conducted in critically ill surgical patients reported that while the predominant admission endotype was “adaptive” for both sepsis and non-sepsis patients, endotypes changed during ICU stay in 57.5% of the population [69]. Patients who remained “adaptive” had a better prognosis, as compared to patients who changed from “adaptive” to “inflammopathic/coagulopathic” [69]. Along the same line, whereas immunotherapy appears to be a highly promising therapeutic approach in sepsis, a major future challenge will be its integration into a clinical setting while guided by easily measurable host response biomarkers that reflect targetable pathophysiological changes driving the sepsis subtype in a time- and individual-dependent manner [15]. Significant challenges remain in translating sepsis subtyping approaches to clinical practice, including reproducibility of omics technologies, bedside tests and computational support, and costs, which will require interdisciplinary collaboration and further technological advancements. Another challenge will be to integrate immunotherapy with other therapeutic options (e.g. resuscitation, antibiotics etc.) to create an optimal personalized therapy for each patient.

## Conclusions

While the current definition of sepsis fails to embody the full spectrum of the syndrome, research seems to be moving in the right direction by attempting to stratify sepsis into subtypes, each treatable by different drugs with specific therapeutic targets. Future research should focus on combining biological and clinical markers with therapeutic options, thereby driving the field of personalized medicine forward within sepsis.
